# Genetic Testing Requires NGS and Sanger Methodologies

**DOI:** 10.15844/pedneurbriefs-30-9-1

**Published:** 2016-09

**Authors:** Lawrence J. Jennings, Dawn Kirschmann

**Affiliations:** 1 Department of Pathology and Laboratory Medicine, Ann & Robert H. Lurie Children’s Hospital of Chicago, Chicago, IL

**Keywords:** NGS, Sanger, SCN1A

## Abstract

Investigators from the EuroEPINOMICS rare epilepsy syndromes Dravet working group performed whole-exome sequencing on 31 trios that had been reported negative for SCN1A mutations by Sanger sequencing.

Investigators from the EuroEPINOMICS rare epilepsy syndromes Dravet working group performed whole-exome sequencing on 31 trios that had been reported negative for SCN1A mutations by Sanger sequencing. They found 8 probands had de novo variants in SCN1A. A survey of 16 genetic centers identified 20 additional probands that had been initially reported as negative by Sanger sequencing but positive by NGS. The investigators present an overview of the reasons for false negative results obtained by Sanger sequencing. Of the 21 cases with a known cause, “human error” was the most common (primarily misread sequence and sample switch). [1]

COMMENTARY. When compared to Sanger sequencing, there are some important advantages to NGS that the investigators mention. Most obviously, NGS allows the acquisition of more sequence data for a lower cost. Mutations in several genes have now been associated with Dravet syndrome (e.g. PCDH19, GABRG2, CHD2, and HCN1), and Sanger sequencing multiple genes can be costly and time-consuming. Also, if trios are performed de novo variants can readily be identified and implicated. This was demonstrated in one intronic variant that was not called initially although seen at the time of Sanger sequencing. NGS data also gives reads on single alleles. Therefore, insertion and deletion variants on the second allele or in the intron do not interfere with the sequence interpretation. This was demonstrated in another case in which an intronic deletion precluded an accurate interpretation of the Sanger sequence data. Also, NGS has the potential to detect mosaicism better than Sanger sequencing which has a limit of detection of 10-20% variant allele [2]. This was demonstrated in a third case in which the mutant peak on Sanger electropherograms were present but too low and misread. Of course, NGS detection of mosaicism depends on the number of reads at that position and the thresholds for calling a variant. NGS sequencing generally is not intended to have the depth to confidently exclude germline mosaicism [3].

This study highlights some problems with Sanger sequencing with the most common error being the misreading of Sanger electropherogram traces by technologists. Reading Sanger sequences can be challenging and requires a high degree of skill and concentration; however, the use of software tools that identify variants for technologist review are available. Such tools can greatly reduce the risk of these types of error. In contrast, poor primer design and sample switch errors are not specific to Sanger sequencing.

We should also note what this study does not tell us. This study is not a comparison of NGS and Sanger sequencing. The authors stated that “our data show that Sanger sequencing resulted in 28 false-negative results while NGS missed one mutation”, which is potentially misleading out of context. By the design of the study, these investigators looked for false-negative results in cases initially screened by Sanger sequencing so understandably that is what they found. A single case that was initially screen positive by Sanger sequencing but negative 8 years later by NGS is included as well, perhaps to highlight a technical limitation of NGS.

Currently, Sanger sequencing is often used to address some of the limitations of NGS such as low-coverage, repetitive sequence, pseudogenes, homopolymer repeats, and large insertions and deletions [3]. These two technologies complement each other and can be used very effectively together. It is important to know what exactly it being sequenced and what are the limitations to each methodology.
